# Outcomes in Single Versus Two-Stage Surgical Stabilization of Complex Bilateral Chest Wall Injury: A Single-Center Experience

**DOI:** 10.7759/cureus.110483

**Published:** 2026-06-08

**Authors:** Alexis A Schweibinz, Jaya S Varre, Anna Graham, William B DeVoe

**Affiliations:** 1 Department of Surgery, Riverside Methodist Hospital (OhioHealth), Columbus, USA

**Keywords:** chest wall injury, chest wall trauma, flail chest, multiple rib fractures, rib fixation, surgical stabilization of rib fractures

## Abstract

Introduction: Surgical stabilization of rib fractures (SSRF) continues to show benefit for severe chest wall injury (CWI) with improvement in morbidity and mortality following stabilization. Complex bilateral injuries pose a clinical and technical challenge. Currently, there is no formal recommendation for an approach to repair such injuries, whether that be bilateral SSRF within the index case or staged repair. We sought to describe our institutional experience with this subset of patients, identify factors that may influence the decision to pursue single- versus two-stage procedures, and assess the potential benefit of combined bilateral SSRF during the index operation.

Methods: A retrospective, single-institution review was conducted at a Level II trauma center including patients undergoing surgical stabilization of bilateral CWI between 2018-2022. Patients were excluded if they received unilateral fixation despite having bilateral CWI or had CWI related to cardiopulmonary resuscitation. Patient/injury characteristics, operative details, and clinical outcomes, including ventilator days, length of stay data, pneumonia, and tracheostomy rates, were analyzed. Descriptive statistics were performed.

Results: Eighteen patients met the inclusion criteria (13 one-stage and 5 two-stage). All patients had flail chest. Patient demographics, overall hospital length of stay (LOS), and post-operative LOS were similar between groups; however, the two-stage group had a higher Injury Severity Score (ISS) (26 one-stage vs 29 two-stage). Total operative time was longer for staged repairs (294 minutes vs 157 minutes for single-stage procedures). The two-stage group required more plates for stabilization (14 vs 8). Patients undergoing staged repair experienced a longer median duration of ventilator dependence (5.5 vs 3 days). Pneumonia was more frequent in the staged group, 40% (n= 2), compared with 8% (n = 1) in the single-stage procedures. Tracheostomy was performed in 40% (n=2) vs 15% (n=2) of patients in the two-stage vs single-stage groups. No inpatient mortality was reported in either cohort.

Conclusion: In this retrospective observational study evaluating outcomes of single and staged SSRF for complex bilateral CWI, expected differences were observed in operative time and number of plates required, both of which were higher in staged repairs. These findings likely reflect increased case complexity and resource utilization associated with staged procedures. Our observational experience highlights the need for careful patient selection and resource planning when considering staged SSRF, with the decision for surgical approach being individualized.

## Introduction

Surgical stabilization of rib fractures (SSRF) continues to show benefit for patients sustaining significant chest wall trauma. Multiple studies have demonstrated improvement in morbidity and mortality following SSRF when patients are carefully selected to undergo surgical management [1-6]. Current guidelines recommend surgical fixation for chest wall instability defined by flail chest, three bi-cortically displaced or offset rib fractures, clinical findings of paradoxical motion, ongoing respiratory failure secondary to chest wall injury, and instability on palpation, amongst other indications [7].

Based on severity and mechanism of injury, complex or bilateral chest wall injury (CWI) is not uncommon. The decision to proceed with surgical intervention in these cases is no different than unilateral chest wall injury and is based on the above criteria, as well as shared decision-making between patients and providers. Most recent studies have indicated that the optimal time for rib fixation is within 72-82 hours of traumatic injury [8,9]. At our institution, unilateral and bilateral chest wall injuries appropriate for operative repair are managed accordingly.

Currently, there is no formal recommendation for an approach to the repair of such injuries, whether that be bilateral SSRF within the index case versus bilateral staged SSRF. Single operation, one-stage bilateral SSRF would entail right and left-sided rib fixation to be completed during the same operation. This could involve two surgeons trained in chest wall stabilization operating on either side of the chest simultaneously or a single surgeon completing the entirety of the operation. A bilateral two-staged intervention intuitively involves two separate operations.

At our institution, both approaches have been performed. It is unclear whether one approach may lead to improved outcomes. To our knowledge, there is limited published literature on this topic. A case report by Patel and Wullschleger was identified, which describes a successful staged approach to bilateral chest wall fixation [10]. With the expansion of our chest wall injury program, we observed a shift in practice to performing single-stage rather than staged chest wall stabilization when able or appropriate in both traumatic and atraumatic (CPR-related) injuries, which also often included cartilage fixation [11,12]. We sought to describe our institutional experience with this subset of patients to identify factors that may influence the decision to pursue single- versus two-stage procedures and assess the potential benefit of combined bilateral SSRF during the index operation.

## Materials and methods

Study design

This retrospective, single-institution exploratory study was conducted at a high-volume Level II trauma center, including patients who underwent surgical stabilization of bilateral chest wall injury (CWI) between 2018 and 2022. The volume of chest wall stabilization cases routinely exceeds 50 procedures annually. Patients were identified through the hospital’s trauma registry and the electronic medical record system. Imaging studies, including chest radiographs and computed tomography (CT) scans, were reviewed to confirm the presence of bilateral rib fractures.

Inclusion and exclusion criteria

Eligible patients were identified from the trauma registry, with inclusion criteria requiring patients to be at least 18 years of age, admitted and discharged between years 2018-2022, and having undergone operative repair of bilateral CWI. Patients were excluded if they sustained a unilateral chest wall injury, did not undergo surgical stabilization of rib fractures (SSRF), or received only unilateral fixation despite having bilateral CWI. We also specifically excluded patients who underwent chest wall stabilization for injuries sustained primarily due to cardiopulmonary resuscitation, as we feel this patient population is unique. As this study was not powered for inferential statistics, the analysis was intended as exploratory in nature.

Data collection

Data was extracted from the trauma registry (Traumabase) and the electronic medical records system. Collected data included demographics (age, sex, medical comorbidities), operative details (total operative time, number of plates implanted, laterality), and clinical outcomes, including hospital and ICU length of stay, ventilator days, and complications, (e.g., pneumonia, tracheostomy). Data quality control was maintained through the use of a standardized data abstraction tool, validation of query parameters, and verification of completeness, duplication, and statistical anomalies.

Outcomes and statistical analysis

The primary outcome was hospital length of stay for patients undergoing single-stage versus two-stage SSRF. Secondary outcomes included ICU length of stay, ventilator days, and complications such as pneumonia and tracheostomy. Descriptive statistics (means, medians, counts, and percentages) were performed to observe trends in the data and to generate hypotheses for testing in larger-scale studies.

## Results

During the study period, a total of 18 patients met the inclusion criteria, with 13 patients undergoing one-stage SSRF and 5 patients undergoing two-stage SSRF. Patient demographics and injury characteristics are summarized in Table 1. The mean age of patients in the one-stage group was 57 years, compared to 61 years in the two-stage group. The Injury Severity Score (ISS) differed between groups, with an ISS of 26 in the one-stage group and 29 in the two-stage group, indicating severe traumatic injury in both cohorts, albeit higher in the patients undergoing staged repair [13]. All patients in both groups had a flail chest. The mean number of fractures was 14 in the one-stage and 19 in the two-stage group.

**Table 1:** Patient demographics and injury characteristics ISS: Injury severity score, N/A: Not applicable, One-stage: A single operation of bilateral surgical rib stabilization, Two-stage: Two separate operations of bilateral surgical rib stabilization.

Characteristic	One-Stage (n=13)	Two-Stage (n=5)
Age, years, mean (SD)	57 (19.4)	61 (14.7)
ISS, mean (SD)	26 (8.9)	29 (0.0)
Number of Rib Fractures, mean (SD)	14 (3.98)	19 (3.42)
Prescence of Flail Chest, n (%)	13 (100)	5 (100)

Operative characteristics highlighted important differences between the groups. The mean time to the operating room (OR) was 78 hours in the one-stage group and 42 hours in the two-stage group (time to first operation). Mean time to OR for the second operation in the two-stage group was 102 hours, again compared to 78 hours in the single-stage group. As anticipated, the total operative time was longer in the two-stage group (Table 2), averaging 294 minutes versus 157 minutes in the one-stage group. The two-stage group required more plates for stabilization, with a mean of 14 plates compared to 8 in the one-stage group.

**Table 2:** Operative characteristics N/A: Not applicable, OR: Operating room, One-stage: A single operation of bilateral surgical rib stabilization, Two-stage: Two separate operations of bilateral surgical rib stabilization.

Characteristic	One-Stage	Two-Stage
Mean Time to OR 1 (hours)	78	42
Mean Time to OR 2 (hours)	N/A	102
Mean Total Case Length (minutes)	157	294
Mean Number of Plates	8	14

Despite differences in operative time and hardware use, the overall hospital length of stay (LOS) was similar between the two groups, with a median of 20 days for the one-stage group and 18 days for the two-stage group (Table 3). The median post-operative LOS was the same in both groups, 16 days. For ICU-related outcomes, the two-stage group had a slightly longer ICU LOS (median 9 vs. 8 days) and mean post-operative ICU LOS (12 vs. 8 days). Patients in the two-stage group experienced a higher incidence of ventilator dependence, with a median of 5.5 ventilator days compared to 3 in the one-stage group. The occurrence of pneumonia was more frequent in the two-stage group, with 40% (n=2) of patients developing it compared with 8% (n=1) in the one-stage group. Tracheostomy was performed in 40% (n=2) of patients in the two-stage group compared to 15% (n=2) in the one-stage group. No inpatient mortality was reported in either group.

**Table 3:** Post operative outcomes ICU: Intensive care unit

Outcome	One-Stage (n =13)	Two-Stage (n=5)
Length of stay, days, median (range)	20 (10-31)	18 (10-40)
Post Operative Length of stay, days, median (range)	16 (6-30)	16 (7-36)
ICU Length of stay, days, median (range)	8 (0-31)	9 (4-34)
Mean Post-Operative ICU length of stay, days	8	12
Ventilator, days, median	3 (0-10)	5.5 (1-13)
Incidence of Pneumonia, n (%)	1 (7.7)	2 (40)
Incidence of Tracheostomy, n (%)	2 (15.4)	2 (40)

## Discussion

The approach to complex and bilateral chest wall injury is challenging, considering the chest wall injury itself, in addition to the frequent and often severe associated traumatic injuries. Based on patient stability and physiology, and given the prioritization of treatment of other injuries, timely stabilization of the chest wall injury is at risk of being delayed beyond the traditional 72-hour threshold [8,9]. The clinical trajectory of this patient population is, as expected, often more complicated than that of patients presenting with unilateral or non-flail fracture patterns [3-5,7]. With the expansion of our chest wall injury program, we have observed a practice pattern shift tending towards single-stage versus two-stage chest wall stabilization depending on the injury pattern, complexity of other associated injuries, and surgeon discretion.

This retrospective observational study describes clinical outcomes following one-stage versus two-stage surgical stabilization of rib fractures (SSRF) in patients with complex bilateral chest wall injuries within our institution. Expected differences were observed in total operative time and the number of plates used, both of which were higher in the two-stage group. The number of rib fractures was also noted to be higher in patients undergoing staged repairs, as was ISS, which likely reflects both greater chest wall injury severity and patient complexity in patients selected for staged intervention. Time to OR was 78 hours for the one-stage group. Time to OR for staged repairs was 42 hours (time to first operation) and 102 hours (time to second operation). This represents, on average, delayed intervention compared to our most recent analysis of institutional time to SSRF (approximately 50 hours) for the single-stage group and the final completion of staged repairs, but still nearing recommendations for fixation within 72-82 hours of presentation [8,9]. The two-stage cohort exhibited higher rates of ICU length of stay, ventilator days, pneumonia, and tracheostomy. These findings may suggest a more complicated initial and postoperative course in this subset of patients, likely influenced by overall injury burden and potentially delayed time to the second operation.

**Figure 1:**
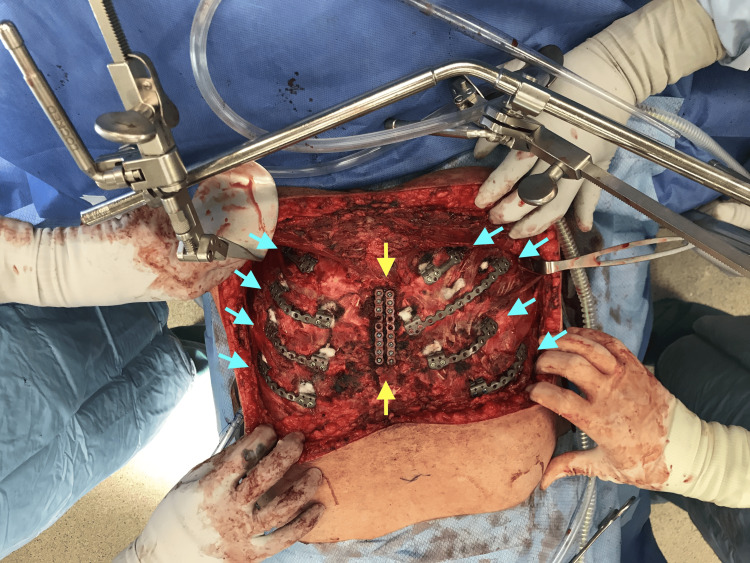
Single stage repair of anterior flail chest wall injury Intraoperative photo of surgical exposure and stabilization of an anterior flail chest wall injury with stabilization of bilateral anterior rib fractures, costal cartilage injuries, and sternal fracture. Blue arrows: stabilization of anterior rib and costal cartilage fractures. Yellow arrows: stabilization of the sternal fracture. The image is from one of the cases treated by the authors.

**Figure 2:**
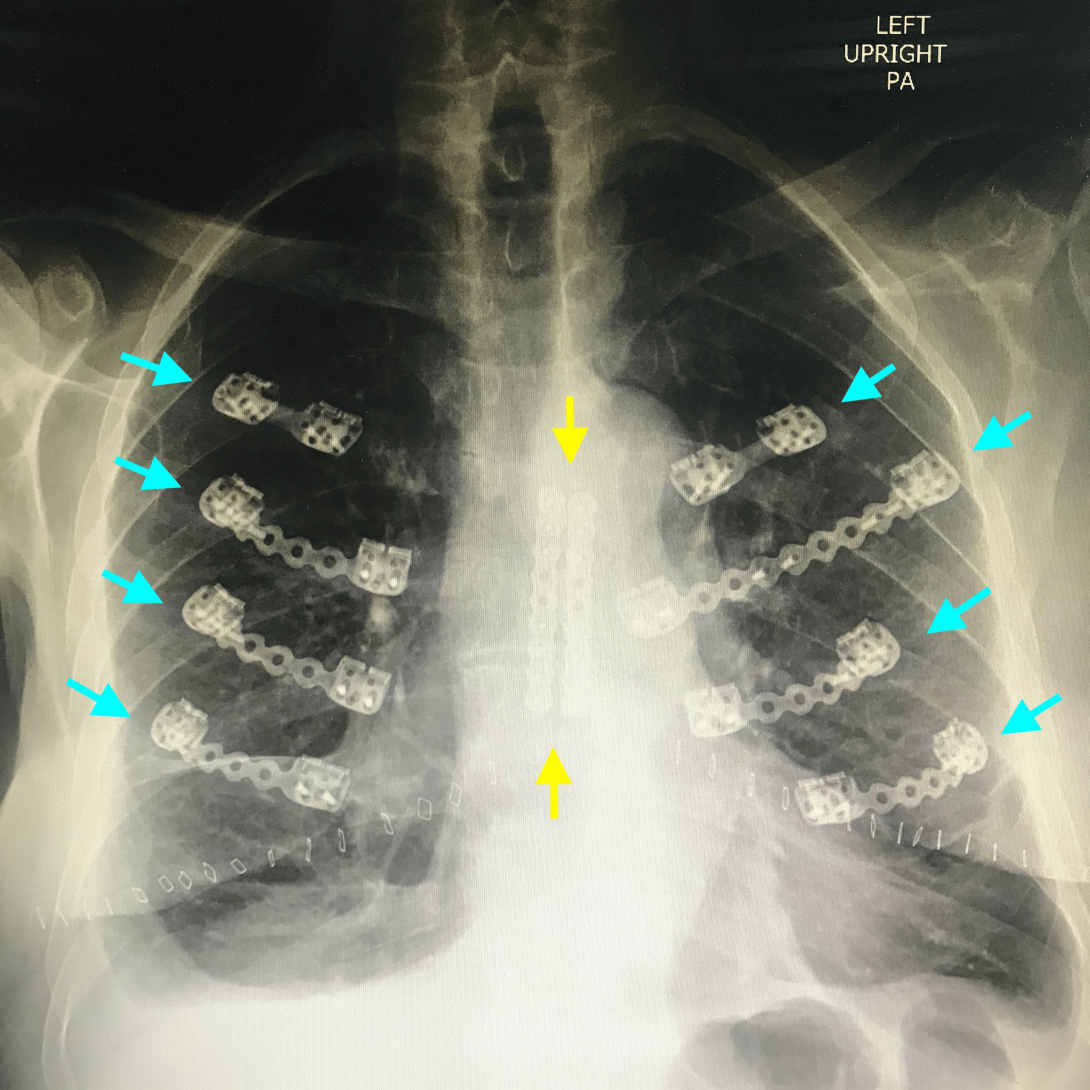
Postoperative radiograph of single stage repair of anterior flail chest wall injury Postoperative radiograph of surgical stabilization of an anterior flail chest wall injury with stabilization of bilateral anterior rib fractures, costal cartilage injuries, and sternal fracture. Blue arrows: stabilization of anterior rib and costal cartilage fractures. Yellow arrows: stabilization of the sternal fracture. The image is from one of the cases treated by the authors.

Our experience demonstrates that the decision between single-stage and staged repair must be individualized, considering the patient’s physiologic reserve, injury burden, and available institutional resources. Management of severe bilateral chest wall injuries is guided by a systematic, multidisciplinary approach within our institution. When feasible, a single-stage repair is coordinated, often utilizing two attending surgeons with expertise in rib fixation to optimize operative efficiency and minimize anesthesia exposure (Figures 1, 2). Increased surgeon capability (>4 trained in SSRF) and improved operating room access with near 24/7 availability for chest wall stabilization have facilitated this approach. In cases with associated spinal or extremity injuries, combined procedures with orthopedic and neurosurgical teams are coordinated to address all injuries in a single operative setting when feasible.

In the literature, there is a paucity of data directly comparing or describing outcomes of single-stage versus staged repairs for this particular subset of patients. Consensus statements and guidelines from the World Society of Emergency Surgery (WSES), Chest Wall Injury Society (CWIS), and American Association for the Surgery of Trauma (AAST) recommend early stabilization of severe chest wall injuries, ideally within 48-72 hours of presentation [14,15]. The WSES-CWIS position paper and WSES-AAST thoracic trauma guidelines do comment on evaluating patients on a case-by-case basis for SSRF when other competing injuries or hemodynamic instability rightfully take priority in the initial days following presentation, while also potentially contributing to delayed (>72 hours) chest wall stabilization. It is often in these scenarios that staged operations are employed, such as that illustrated in a case report by Patel et al. describing a successful staged bilateral chest wall fixation for complex CWI and lung herniation [10]. The authors followed damage control principles to perform anterior chest wall stabilization during a period of cardiopulmonary compromise, 10 hours after patient presentation, followed by stabilization of the left posterolateral fractures 48 hours later when the patient's cardiopulmonary status had stabilized. The postoperative course was notable for tracheostomy placement and prolonged hospital stay, similar to several cases of staged repair observed in the study herein. 

Given the limited sample size and variability in patient characteristics, we are unable to draw definitive conclusions regarding the optimal operative strategy. This pilot-type study is limited by its retrospective, single-institution design and small sample size, which constrain both the ability to provide meaningful statistical analysis and generalizability. Additionally, an institutional preference for one-stage SSRF when feasible may introduce selection bias. The specific chest wall injury characteristics and patient-related factors were varied amongst each cohort, while also being limited in detail, given the dataset, which limits the impact on patient selection for single versus staged repair. A deeper analysis of the patients treated herein and in the years following completion of the current pilot study is planned. The absence of long-term functional or quality-of-life outcomes also limits the assessment of the broader impact of surgical staging on recovery. Given these limitations and the small sample size, our findings should be interpreted with caution, highlighting the need for larger-scale or multi-center evaluations to assess the influence of staged repair on patient outcomes.

## Conclusions

In this retrospective single-center observational study describing outcomes of single-stage versus two-stage surgical stabilization of rib fractures (SSRF) in bilateral chest wall injuries, expected differences were observed in total operative time and the number of plates used, both of which were higher in the two-stage group. These findings likely reflect increased case complexity and resource utilization associated with staged procedures. Although other outcomes, such as ICU length of stay, ventilator days, pneumonia, and tracheostomy, were more frequent in the two-stage cohort, definitive comparative analysis was not performed due to the limitations of the small sample size and patient variability. These findings highlight the need for careful patient selection and resource planning when considering staged SSRF, with the decision for surgical approach being individualized for patients with complex or bilateral chest wall injuries.
